# Inflammation mediated the effect of dietary fiber on depressive symptoms

**DOI:** 10.3389/fpsyt.2022.989492

**Published:** 2023-01-11

**Authors:** Ting Zhang, Xiao-mei Cui, Yu-ying Zhang, Tao Xie, Yuan-jia Deng, Fang-xi Guo, Qi Wang, Qing-feng Wu, Ming-hua Dong, Xiao-ting Luo

**Affiliations:** ^1^Key Laboratory of Cardiovascular and Cerebrovascular Diseases, Ministry of Education, Gannan Medical University, Ganzhou, China; ^2^School of Public Health and Health Management, Gannan Medical University, Ganzhou, China; ^3^Department of Epidemiology, Gannan Medical University, Ganzhou, China; ^4^Department of General Practice, Gannan Medical University, Ganzhou, China

**Keywords:** depression, nutrition, dietary fiber, dietary inflammatory index, CRP

## Abstract

**Background and aims:**

Previous studies showed that inflammation affects depressive symptoms. Dietary fiber may be associated with inflammation and depressive symptoms. We aimed to investigate the relationship between inflammation and depressive symptoms at different levels of dietary fiber intake and to explore whether dietary fiber affects depression through inflammation.

**Methods:**

A total of 8,430 National Health and Nutrition Examination Survey (NHANES) samples were collected between 2015 and 2018. Factor analysis was used to determine dietary patterns. Linear regression and logistic regression analysis were used to explore the relationship between nutrients, inflammation, and depressive symptoms, and the mediation analysis was conducted using the bootstrap method.

**Results:**

Factor 3 (dietary fiber and vitamins) was inversely associated with depressive symptoms and inflammation. The upper quartile scores of the dietary inflammatory index (DII) and C-reactive protein (CRP) were associated with depressive symptoms compared with controls (DII: *OR* = 1.851, 95% CI: 1.267–2.705; CRP: *OR* = 1.737, 95% CI: 1.136–2.656). The DII score and CRP were associated with depressive symptoms in the group with low dietary fiber intake (DII: *OR* = 2.736, 95% CI: 1.628–4.598; CRP: *OR* = 2.092, 95% CI: 1.196–3.658) but not in the high dietary fiber intake group. Mediating analysis showed that CRP partially mediated the effect of dietary fiber intake on depressive symptoms (β_indirect_ = −0.0025, 95% CI: −0.0038 to −0.0013), and the mediated proportion was 10.5%.

**Conclusion:**

In this study, we found that DII scores and CRP were not associated with depressive symptoms in participants with high dietary fiber intake, and inflammation partially mediates the effect of dietary fiber on depressive symptoms.

## Introduction

Depressive symptoms were one of three major causes of global years lived with disability (YLD) for women in 2017 (1). They are projected to be the leading cause of the global burden of disease by 2030 (2). Major depressive symptoms have a high prevalence in the United States and are a widespread and serious health problem; the lifetime and 12-month prevalence of major depressive symptoms were 20.6 and 10.4%, respectively (3), and the majority of Americans with depressive symptoms do not receive treatment (4). Depressive symptoms, as a psychological disease, bring serious burdens to individuals and society. They frequently reoccur, and the longer they persist, the worse the prognosis. In addition, depressive symptoms can lead to suicidal tendencies (5–7) and are also strongly associated with cardiovascular disease (8, 9). Therefore, identifying the relevant factors of depressive symptoms plays an important role in the prevention and management of depressive symptoms.

Unhealthy diets are a significant contributor to the Global Burden of Disease (GBD), with a 2017 GBD analysis showing that diets contribute to 11 million deaths and 255 million disability-adjusted life years (10). Dietary consumption is continually changing as industrialization and globalization progresses. Some studies showed that nutritionally unbalanced diets, such as those based on ultra-processed foods, are harmful to health (11). In a prospective study of 5.4 years, a 10% increase in the proportion of ultra-processed foods in the diet was associated with a 1.21-fold increased risk of depressive symptoms (12). Numerous studies linked nutrients such as zinc, dietary fiber, folic acid, and vitamins in the serum to depressive symptoms (13–17). Balanced and healthy nutrient intake plays an important role in reducing inflammation and oxidative stress (18). Nutrients are associated with inflammation, and the dietary inflammation index was developed to represent the anti-inflammatory or pro-inflammatory nature of the diet of an individual (19). Studies linked dietary inflammatory index (DII) score to depressive symptoms and inflammation (20, 21).

The pathogenesis of depressive symptoms is closely associated with inflammation (22). In recent years, studies found that dietary fiber is associated with depressive symptoms (23). Dietary fiber may reduce inflammation by altering the intestinal microenvironment (24), and decreased inflammation reduces the effects of neurotransmitters on depressive symptoms (22, 25). We hypothesized that nutritional fiber consumption would possibly have an effect on depressive symptoms *via* inflammation and that higher nutritional fiber consumption would possibly reduce inflammation, leading to an altered association between inflammation and depressive symptoms. However, few studies examined the association of inflammation with depressive symptoms in participants with different intakes of dietary fiber. Therefore, we used data from the 2015–2018 National Health and Nutrition Examination Survey (NHANES) to explore the associations between inflammation and depressive symptoms within the levels of dietary fiber intake and whether dietary fiber affects depression through inflammation. In future, it is expected to regulate the level of inflammation by changing dietary fiber intake and ultimately prevent depressive symptoms.

The purpose of this study was to investigate the association of nutritional intake with depressive symptoms and inflammation among U.S. residents from 2015 to 2018 and to explore the distribution of DII scores in patients with different degrees of depressive symptoms. Logistic regression was used to analyze the_association of DII and C-reactive protein (CRP) quartile scores with depressive symptoms in total participants and participants with different dietary fiber intakes and to further explore whether dietary fiber influences depression through inflammation.

## Methods

### Study design of NHANES 2015–2018

The National Health and Nutrition Examination Survey is one of a series of health-related projects conducted by the National Center for Health Statistics (NCHS) to provide information on the health and nutrition status of non-hospital residents in the United States (26). Approximately 5,000 nationally representative participants are sampled annually through multistage, complex, stratified, and cluster sampling methods. The details of NHANES 2015–2018 have been reported in previous studies (27). We used data from NHANES from the 2015–2016 and 2017–2018 survey cycles (28, 29). The NHANES research was approved by the NCHS Research Ethics Review Board. All participants provided written informed consent.

### Study participants

We collected 9,972 individual records in the NHANES 2015–2016 survey cycle and 9,255 individual records in the NHANES 2017–2018 survey cycle. We excluded participants under the age of 18 (*n* = 7,377) and those with an incomplete dietary and depressive record (*n* = 3,420). Finally, 8,430 samples were included.

### Exposures

#### Nutrient measurements

Dietary intake data were obtained by estimating the type and amount of food and drink consumed in the 24-h dietary recall interviews and by estimating the energy (28, 29), nutrients, and other food components consumed from these foods and drinks. The Food Surveys Research Group (FSRG) of the USDA is responsible for dietary data collection methodology (30). All NHANES participants attended two 24-h dietary recall interviews. The first dietary recall interview was collected in person at the Mobile Examination Center (MEC), and the second interview was collected by telephone 3–10 days later. We extracted the following 30 variables from the nutrient intake information: energy, protein, carbohydrate, dietary fiber, total fat, saturated fatty acid (SFA), monounsaturated fatty acids (MUFA), polyunsaturated fatty acids (PUFA), cholesterol, vitamin E, retinol, vitamin A, vitamin B_1_, vitamin B_2_, niacin, vitamin B_6_, folic acid, total choline, vitamin B_12_, vitamin C, vitamin D, vitamin K, calcium, iron, zinc, copper, sodium, potassium, alcohol, and caffeine.

#### DII

The DII score (19), which describes levels of dietary inflammation, was originally developed from 45 dietary variables. We extracted 22 nutrients (alcohol, vitamin B_12_, vitamin B_6_, caffeine, carbohydrate, cholesterol, energy, total fat, dietary fiber, folic acid, iron, MUFA, PUFA, SFA, niacin, protein, vitamin A, vitamin C, vitamin D, vitamin E, zinc, and vitamin B_2_) to calculate DII. DII was calculated by subtracting the global average intake from the extracted nutrient level and dividing it by the standard deviation of the global average intake (19). This value was converted to a centered percentile score to minimize the effect of “right skew.” The centered percentile for each nutrient was multiplied by its respective inflammation score (19), and these new values were finally added to obtain the overall DII index for each participant. Higher DII scores indicate higher levels of dietary inflammation. Lower DII scores indicate lower levels of dietary inflammation.

### Outcomes

#### Depressive symptoms

We administered the Patient Health Questionnaire (PHQ-9) to determine the frequency of depressive symptoms over the past 2 weeks (31). For each question, the scores ranged from 0 to 3 for “none at all”, “several days”, “more than half”, and “almost every day.” Scores can range from 0 to 27. Higher scores were associated with a higher risk of depressive symptoms. A PHQ-9 score range of 5 to 9 indicates mild depressive symptoms, 10 to 14 indicates moderate depressive symptoms, 15 to 19 indicates moderately severe depressive symptoms, and 20 to 27 indicates severe depressive symptoms (31).

### Sociodemographic characteristics

Sociodemographic characteristics included age, gender, race, days of vigorous recreational activities, minutes of sedentary activity, systolic blood pressure (SBP), diastolic blood pressure (DBP), body mass index (BMI), CRP, serious difficulty hearing, serious difficulty seeing, serious difficulty concentrating, and taking medication for depressive symptoms.

All SBP and DBP measurements were taken at the MEC. After resting quietly in a seated position for 5 min, three consecutive BP measurements (systolic and diastolic) were taken 60 s apart using a digital upper-arm electronic blood pressure measurement device, the Omron HEM−907XL. In our study, we used the mean of the readings to define the final blood pressure. The data on the body measurement were collected at the Mobile Examination Center (MEC) by trained health technicians: BMI = (weight)/(height)^2^. “Minutes of sedentary activity” is defined as time spent sitting at a desk, traveling in a car, reading, playing cards, watching TV, using the computer, and so on; however, time spent sleeping is not included. “Vigorous recreational activities” is defined as a large increase in breathing or heart rate that is done for at least 10 min continuously. The participants were also asked the following questions: Do you have severe difficulty hearing? Do you have serious difficulty seeing even when wearing glasses? Do you have serious trouble concentrating, remembering things, or making decisions?

### Statistical analysis

We used means, standard deviation (*SD*), and percentages to describe the basic characteristics of the groups with depressive symptoms and non-depressive symptoms. The *T*-test was used for numerical variables, and the chi-square test was used for categorical variables. Simple linear regression and multiple linear regression were used to find dietary nutrition variables associated with the PHQ-9. After converting nutrients into *Z*-scores, radar and heat maps were created to describe the distribution of nutrients among varying levels of depressive symptoms and non-depressive symptoms. We simplified the 28 nutrient variables into 4 factors through factor analysis and performed multiple linear regression with the PHQ-9 and CRP. The Kaiser-Meyer-Olkin (KMO) test is used to verify the applicability of factor analysis, and a KMO value exceeding 0.7 is considered suitable for factor analysis. Correlation between nutrients was tested using Bartlett's test of sphericity. A *P*-value of <0.05 is acceptable (32). Principal component analysis was used to extract factors and orthogonal rotation (varimax option) to derive uncorrelated factors (33). We obtained a total of four factors and calculated a score for each of the four factors for each participant. Multivariate logistic regression was used to estimate odds ratios (*OR*) and 95% confidence intervals (*CI*) between the DII index, CRP, and depressive symptoms (PHQ-9 score >15, moderate-severe, and severe depressive symptoms). We adjusted the models for confounding factors: model 2 adjusted for sex, age, and race; model 3 adjusted for vigorous exercise, minutes of sedentary time, SBP, DBP, and BMI based on model 2; and model 4 continued to adjust for serious difficulty concentrating, serious difficulty hearing, serious difficulty hearing, and depression medication based on model 3.

### Mediation analysis

The SPSS PROCESS plug-in was used for mediation analysis, with dietary fiber as the independent variable, the depressive symptoms score (PHQ-9) as the dependent variable, and CRP as the intermediate variable. Pathway (c) represents the total effect of exposure factors (dietary fiber) on outcome variables (PHQ-9), pathway (c') represents the direct effect of exposure variables on outcome variables, pathway (a) represents the impact of exposure variables on mediators, and pathway (b) represents the impact of mediators on outcome variables. The proportion of the mediated effect was calculated using the following formula: (indirect effect/total effect) × 100%. Statistical analysis was performed using SPSS 23.0 (IBM SPSS, Inc., Chicago, IL, USA). A *P*-value of <0.05 was considered statistically significant.

### Sensitivity analysis

We hypothesize that dietary patterns affect depressive symptoms, but it is reasonable that depressive symptoms also have an effect on dietary patterns. Therefore, we conducted sensitivity analyses using linear regression to investigate the association of depressive symptoms with dietary patterns and adjusted for the abovementioned confounding factors. In addition, acute inflammation may affect the association between dietary fiber intake and CRP with depressive symptoms. We did a sensitivity analysis by excluding participants with acute inflammation.

## Results

The basic sociodemographic characteristics of this study are shown in Table 1. Among the participants with non-depressive symptoms and depressive symptoms, those aged over 50 years (51.8, 54.3%), non-Hispanic whites (34.9, 39.0%), those with serious difficulty hearing (7.4, 14.6%), those with serious difficulty seeing (4.2, 11,9%), and those with serious difficulty concentrating (4.1, 29.0%) accounted for a large proportion in participants with depressive symptoms. The majority of patients who are depressed are women, while the majority of patients who are not depressed are men; the difference was statistically significant (*P* < 0.05). BMI, CRP, and minutes of sedentary time were higher in participants with depressive symptoms than in participants with non-depressive symptoms (*P* < 0.05).

**Table 1:** Basic characteristics of the participants.

**Variables**	**Non-depressive symptoms** ** (*n* = 6,252)**	**depressive symptoms** ** (*n* = 2,177)**	***P*-value**
**Demographic factor [*****n*** **(%)]**			
Age (years) (categorical)			
18–50	3,011 (48.2)	994 (45.7)	0.044
Over 50	3,241 (51.8)	1,183 (54.3)	
**Marital status**			
Married	3,201 (51.2)	1,085 (49.8)	0.263
Single	3,047 (48.8)	1,096 (50.2)	
**Race**			
Mexican American	995 (15.9)	336 (15.4)	< 0.001
Other Hispanic	680 (10.9)	268 (12.3)	
Non-Hispanic white	2,184 (34.9)	849 (39.0)	
Non-Hispanic black	1,347 (21.5)	458 (21.0)	
Other Race–Including multi-racial	1,046 (16.7)	266 (12.2)	
**Gender**			
Men	3,230 (51.7)	897 (41.2)	< 0.001
Women	3,022 (48.3)	1,280 (58.8)	
**Serious difficulty hearing**			
Yes	465 (7.4)	317 (14.6)	< 0.001
No	5,787 (92.6)	1,860 (85.4)	
**Serious difficulty seeing**			
Yes	263 (4.2)	259 (11.9)	< 0.001
No	5,989 (95.8)	1,918 (88.1)	
**Serious difficulty concentrating**			
Yes	256 (4,1)	631 (29.0)	< 0.001
No	5,996 (95.9)	1,546 (71)	
**Vigorous exercise**			
Yes	1,678 (26.8)	407 (18.7)	< 0.001
No	4,574 (73.2)	1,770 (81.3)	
**Demographic factor (mean** ±**SD)**			
SBP (mm Hg)	125.7 ± 18.46	126.19 ± 19.36	0.396
DBP (mm Hg)	70.80 ± 13.07	70.93 ± 13.02	0.697
CRP (mg/L)	3.74 ± 6.22	5.42 ± 10.86	< 0.001
BMI (kg/m^2^)	29.47 ± 6.89	30.97 ± 7.97	< 0.001
Minutes of sedentary time (minutes)	347.28 ± 197.80	369.00 ± 212.94	< 0.001

Patients who are depressed had higher intakes of vitamin B_6_ and saturated fatty acids than participants who are not depressed, and lower intakes of dietary fiber, folic acid, vitamin C, vitamin K, and zinc than participants who are not depressed. Among patients who are depressed, the intake of vitamin B6 was higher in patients with major depressive symptoms than in those with other depressive symptoms, but the intake of protein, dietary fiber, MUFA, PUFA, cholesterol, vitamin E, folic acid, choline, and iron was lower in patients with the major depressive disorder than in those with other depressive symptoms (Supplementary Figures 1, 2).

### Association of nutritional composition with depressive symptoms

The results of the simple linear regression between nutrient intake and the PHQ-9 score of participants are shown in Table 2. Protein, dietary fiber, PUFA, vitamin E, vitamin B1, folate, choline, vitamin C, vitamin K, iron, zinc, copper, sodium, potassium, and caffeine intake were associated with the PHQ-9 scores (*P* < 0.05). Multiple linear regression results showed that vitamin E and caffeine were positively correlated with the PHQ-9 scores (*P* < 0.05), and protein, dietary fiber, total folate, and copper were negatively correlated with the PHQ-9 scores (*P* < 0.05) (Supplementary Table 1).

**Table 2:** Simple linear regression between nutrient intake and PHQ-9 score in the participants.

**PHQ-9**			**PHQ-9**		

**Variables**	**Standardized Coef**.	***P*** **-value**	**Variables**	**Standardized Coef**.	***P*** **-value**
Energy	−0.007	0.532	Total folate	−0.063	**< 0.001**
Protein	−0.043	**< 0.001**	Total choline	−0.033	**0.002**
Carbohydrate	0.001	0.963	Vitamin B12	−0.003	0.791
Dietary fiber	−0.075	**< 0.001**	Vitamin C	−0.034	**0.002**
Total fat	−0.004	0.681	Vitamin D	−0.013	0.233
SFA	0.013	0.240	Vitamin K	−0.037	**0.001**
MUFA	−0.008	0.460	Calcium	−0.017	0.110
PUFA	−0.022	**0.042**	Iron	−0.045	**< 0.001**
Cholesterol	−0.017	0.133	Zinc	−0.030	**0.006**
Vitamin E	−0.025	**0.022**	Copper	−0.058	**< 0.001**
Retinol	−0.001	0.924	Sodium	−0.023	**0.032**
Vitamin A	−0.018	0.093	Potassium	−0.051	**< 0.001**
Vitamin B1	−0.033	**0.002**	Caffeine	0.04	**< 0.001**
Vitamin B2	−0.010	0.381	Alcohol	0.010	0.358
Niacin	−0.010	0.359	Moisture	0.002	0.830
Vitamin B6	0.0001	0.988			

Bold values indicate that nutrients are associated with depressive symptoms.

### Factor rotation of nutrient intake variables and multiple linear regression with the PHQ-9 scores and CRP

The score of 28 nutrients through factor rotation is shown in Table 3. Total fat, MUFA, SFA, energy, PUFA, sodium, cholesterol, protein, choline, and carbohydrates had the highest scores in factor 1. Vitamin B_1_, niacin, vitamin B_2_, zinc, iron, vitamin B_6_, and vitamin B_12_ had the highest scores in factor 2. Potassium, vitamin E, copper, dietary fiber, folic acid, vitamin K, and vitamin C scored highest in factor 3, and calcium, vitamin D, vitamin A, and retinol scored highest in factor 4.

**Table 3:** The result of factor rotation.

	**Factor 1**	**Factor 2**	**Factor 3**	**Factor 4**
Total fat	**0.921**	0.176	0.214	0.115
MUFA	**0.895**	0.175	0.199	0.085
SFA	**0.831**	0.186	0.108	0.211
Energy	**0.794**	0.409	0.326	0.069
PUFA	**0.787**	0.072	0.309	−0.016
Sodium	**0.735**	0.365	0.229	0.08
Cholesterol	**0.682**	0.112	−0.131	0.447
Protein	**0.673**	0.482	0.162	0.267
Total choline	**0.647**	0.301	0.075	0.444
Carbohydrate	**0.505**	0.434	0.469	−0.056
Vitamin B1	0.381	**0.572**	0.436	0.155
Niacin	0.37	**0.816**	0.107	0.08
Vitamin B2	0.346	**0.63**	0.203	0.387
Zinc	0.34	**0.551**	0.146	0.304
Iron	0.314	**0.553**	0.49	0.239
Vitamin B6	0.11	**0.796**	0.116	0.114
Vitamin B12	0.095	**0.69**	−0.06	0.49
Potassium	0.452	0.389	**0.582**	0.223
Vitamin E	0.448	0.152	**0.541**	0.142
Copper	0.275	0.375	**0.459**	0.237
Dietary fiber	0.262	0.261	**0.759**	−0.038
Total folate	0.205	0.497	**0.607**	0.178
Vitamin K	0.073	−0.161	**0.561**	0.196
Vitamin C	0.011	0.078	**0.618**	0.093
Calcium	0.408	0.269	0.357	**0.441**
Retinol	0.145	0.214	0.193	**0.775**
Vitamin D	0.122	0.218	0.067	**0.626**
Vitamin A	0.071	0.078	0.469	**0.717**

Bold values indicate factor loading of greater than 0.4.

After multivariate adjustment, factor 3 was negatively associated with depressive symptoms and CRP (Table 4). In addition, sensitivity analysis showed that depressive symptoms were negatively correlated with factor 3 (Supplementary Table 2).

**Table 4:** Multiple linear regression of 4 factors with PHQ-9 scores and CRP.

**Variable**	**PHQ-9 scores**		**CRP**	

	**Standardized Coef**.	***P*** **-value**	**Standardized Coef**.	***P*** **-value**
Factor 1	0.019	0.061	−0.018	0.094
Factor 2	0.012	0.245	−0.004	0.718
Factor 3	−0.028	**0.004**	−0.049	**< 0.001**
Factor 4	−0.004	0.704	−0.019	0.064

We used age, sex, race, vigorous exercise, minutes of sedentary time, SBP, DBP, BMI, serious difficulty concentrating, serious difficulty hearing, serious difficulty seeing, and depression medication as adjusted variables in multiple linear regression. Bold values denote statistical significance with *p*-values <0.05.

### Effects of DII scores and CRP on depressive symptoms in the total participants and different dietary fiber subgroups

We used the *T*-test to analyze differences in DII scores between patients who are depressed and those who are not depressed in the total participants. There were differences in DII scores between non-depressive symptoms and depressive symptoms of different degrees among all participants (*P* < 0.05) (Supplementary Figure 3).

Table 5 shows the correlation between DII scores and CRP on depressive symptoms (moderate and severe depressive symptoms) in total participants and different dietary fiber intake groups. The median dietary fiber intake was calculated. Those below the median were defined as a low dietary fiber intake group, while those above the median were defined as a high dietary fiber intake group. With Q_1_ as the reference group, the Q_4_ group of DII scores was significantly associated with depressive symptoms, and the degree of association was constant after the addition of adjusted variables (*OR* = 1.851, 95% CI: 1.267–2.705). In the subgroup with low dietary fiber intake, the upper quartile of DII scores was significantly associated with depressive symptoms compared with the control group (*OR* = 2.736, 95% CI: 1.628–4.598) and the degree of association increased. DII scores were not significantly associated with depressive symptoms in the subgroup with high dietary fiber intake. The CRP of the Q_4_ group was significantly associated with depressive symptoms, and the degree of association was constant after adjusting for variables (*OR* = 1.737, 95% CI: 1.136–2.656). In the subgroup with low dietary fiber intake, the upper quartile of CRP was significantly associated with depressive symptoms compared with the control group (*OR* = 2.092, 95% CI: 1.196–3.658), and there was no correlation in the high dietary fiber intake group. Sensitivity analyses showed that, when participants with acute inflammation (CRP ≥ 10) were excluded, the association between CRP and depressive symptoms was consistent with previous results, indicating that our results were robust (Supplementary Table 3).

**Table 5:** *OR* and 95% CI for depressive symptoms (moderately severe and severe depressive symptoms) according to DII scores and CRP in the entire group and different dietary fiber intake groups.

	**Model 1**	**Model 2**	**Model 3**	**Model 4**
**DII, Entire group**				
Q_1_ (≤ −0.945)	1.000	1.000	1.000	1.000
Q_2_ (> −0.945 to −0.007)	1.359 (0.924–2.000)	1.347 (0.915–1.982)	1.309 (0.888–1.930)	1.271 (0.844–1.913)
Q_3_ (> −0.007 to 0.934)	1.314 (0.891–1.939)	1.311 (0.888–1.934)	1.229 (0.830–1.820)	1.096 (0.725–1.656)
Q_4_ (>0.934)	2.445 (1.723–3.470)	2.520 (1.774–3.579)	2.379 (1.665–3.400)	1.851 (1.267–2.705)
**DII, Lower dietary fiber intake**				
Q_1_ (≤ 0.160)	1.000	1.000	1.000	1.000
Q_2_ (>0.160 to 0.860)	1.401 (0.814–2.410)	1.385 (0.805–2.386)	1.376 (0.798–2.372)	1.555 (0.876–2.762)
Q_3_ (>0.860 to 1.530)	1.663 (0.984–2.812)	1.645 (0.912–2.784)	1.668 (0.982–2.823)	1.715 (0.979–3.002)
Q_4_ (>1.530)	3.274 (2.029–5.282)	3.274 (2.023–5.300)	3.310 (2.041–5.368)	2.736 (1.628–4.598)
**DII, More dietary fiber intake**				
Q_1_ (≤ -1.480)	1.000	1.000	1.000	1.000
Q_2_ (>−1.480 to −0.840)	1.453 (0.803–2.630)	1.424 (0.786–2.579)	1.434 (0.790–2.662)	1.478 (0.787–2.775)
Q_3_ (>−0.840 to −0.180)	1.900 (1.080–3.344)	1.766 (1.001–3.115)	1.754 (0.992–3.101)	1.911 (1.045–3.495)
Q_4_ (>−0.180)	1.731 (0.975–3.073)	1.552 (0.869–2.770)	1.489 (0.831–2.665	1.317 (0.710–2.448)
**CRP** **(mg/L), Entire group**				
Q_1_ (≤ 0.840)	1.000	1.000	1.000	1.000
Q_2_ (>0.840 to 2.000)	1.163 (0.775–1.746)	1.118 (0.744–1.679)	1.069 (0.708–1.615)	1.056 (0.684- 1.629)
Q_3_ (>2.000 to 4.600)	1.836 (1.262–2.670)	1.753 (1.203–2.553)	1.594 (1.078–2.358)	1.456 (0.964–2.201)
Q_4_ (>4.600)	2.442 (1.707–3.495)	2.339 (1.632–3.351)	2.020 (1.357–3.008)	1.737 (1.136–2.656)
**CRP** **(mg/L), Lower dietary fiber intake**				
Q_1_ (≤ 0.910)	1.000	1.000	1.000	1.000
Q_2_ (>0.910 to 2.300)	1.421 (0.847–2.384)	1.329 (0.789–2.237)	1.323 (0.781–2.241)	1.327 (0.757–2.328)
Q_3_ (>2.300 to 5.230)	1.535 (0.917–2.569)	1.431 (0.852–2.403)	1.368 (0.800–2.337)	1.291 (0.730–2.282)
Q_4_ (>5.230)	2.752 (1.723–4.396)	2.562 (1.596–4.114)	2.397 (1.431–4.015)	2.092 (1.196–3.658)
**CRP, mg/L More dietary fiber intake**				
Q_1_ (≤ 0.800)	1.000	1.000	1.000	1.000
Q_2_ (>0.800 to 1.720)	0.821 (0.429–1.572)	0.792 (0.412–1.521)	0.739 (0.383–1.482)	0.694 (0.348–1.383)
Q_3_ (>1.720 to 4.000)	1.711 (0.997–2.937)	1.591 (0.921–2.746)	1.438 (0.816–2.536)	1.280 (0.700–2.339)
Q_4_ (>4.000)	1.979 (1.168–3.353)	1.741 (1.019–2.976)	1.510 (0.832–2.739)	1.301 (0.693–2.441)

Model 1: not adjusted. Model 2: adjusted for age, sex, and race. Model 3: adjusted for variables in model 2 + vigorous exercise, minutes of sedentary time, SBP, DBP, and BMI. Model 4: adjusted for variables in model 3 + serious difficulty concentrating, serious difficulty hearing, serious difficulty seeing, and depression medication.

### Mediating analysis of dietary fiber, CRP, and depressive symptoms

**Figure 1:**
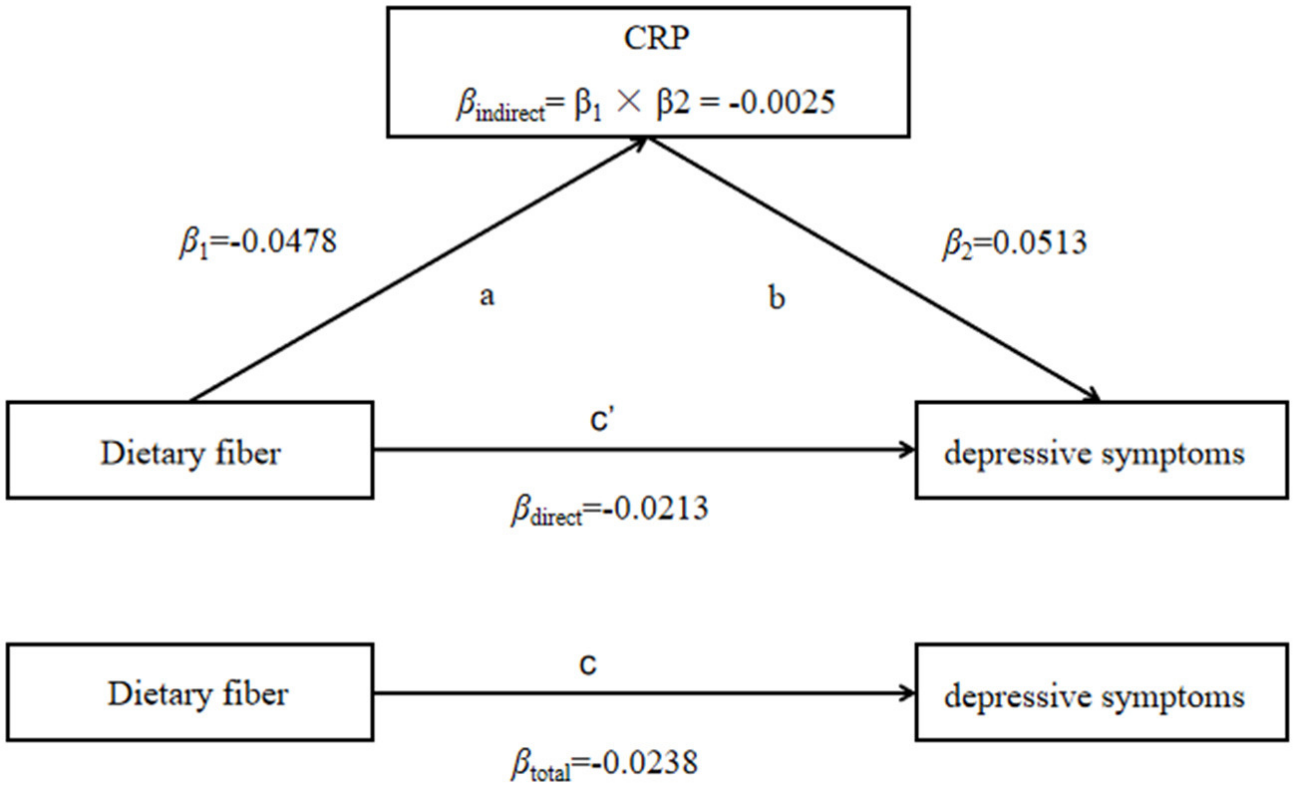
CRP mediated the association between dietary fiber intake and depressive symptoms (PHQ-9 score as continuity variable), path ab represents the indirect effect, path c represents the direct effect, and path c represents the total effect.

The correlation between inflammation and depressive symptoms varied across stratified analyses of dietary fiber intake, so we further explored whether CRP for the intermediary role in the process of dietary fiber intake influences depressive symptoms. The results showed that CRP partially mediates the effect of dietary fiber on depressive symptoms (β_indirect_ = −0.0025, 95% CI: −0.0038 to −0.0013), and the mediated proportion was 10.5% (Table 6, Figure 1).

**Table 6:** Mediating analysis of dietary fiber, CRP, and depressive symptoms.

	**Estimate**	**95% CI**
Total effect	−0.0238	−0.0366 to −0.0110
Direct effect	−0.0213	−0.0341 to −0.0086
Indirect effect	−0.0025	−0.0038 to −0.0013
Path a	−0.0478	−0.0709 to −0.0247
Path b	0.0513	0.0369 to 0.0631

Adjusted for factor 3 (dietary pattern dominated by dietary fiber and vitamins).

## Discussion

This study provides evidence that dietary fiber, CRP, and DII scores are associated with depressive symptoms. Protein, dietary fiber, folic acid, and copper intake were negatively correlated to PHQ-9 scores, while vitamin E and caffeine intake were positively correlated with PHQ-9 scores. Dietary patterns dominated by dietary fiber and vitamins were negatively correlated with depression and CRP. We showed that dietary fiber is associated with depression and inflammation. DII scores as an indicator of dietary inflammation were significantly associated with depressive symptoms, with an increased association among participants with low dietary fiber intake but no significant association among participants with high dietary fiber intake. As with DII results, the CRP levels were significantly associated with depressive symptoms, but there was no significant association among participants with high dietary fiber intake. Finally, we further demonstrated that CRP plays a partial mediating role in the effect of dietary fiber on depressive symptoms.

Previous studies explained a significant association between folic acid levels and depressive symptoms (34, 35). Protein consumption was associated with a lower risk of depressive symptoms (36), and higher dietary fiber intake was inversely associated with depressive symptoms (37, 38). Li et al. used NHANES data from 2009 to 2014 to show that copper is inversely related to depressive symptoms, which is consistent with the results of our study (39). In contrast to our study, a meta-analysis of 25 observational studies showed an inverse association between vitamin E and depressive symptoms (40). Second, our study showed that, after adjusting for a variety of nutrients, caffeine consumption is positively associated with depressive symptoms. However, caffeine consumption was inversely associated with the prevalence of self-reported lifetime depression among Koreans (41), and future research needs to be designed to observe the potential benefits of caffeine.

Factor 3, mainly vitamins and dietary fiber, was negatively correlated with depressive symptoms and CRP, with the same results in the entire group, the >50-year-old subgroup, and the male subgroup. A meta-analysis showed that vitamin C intake was inversely associated with depressive symptoms (40), and that tocopherol treatment prevented TNF-α-induced depressive symptoms in mice (42). In addition, studies showed that oxidative stress in patients with depressive symptoms increases (43, 44), and the intake of vitamin C, vitamin E, and folic acid is related to a reduction in oxidative stress (45–47). Vitamin C has the potential to prevent and treat depressive symptoms because it protects neurons from oxidative stress and reduces inflammation (48). Folic acid treatment in mice restored the activity of antioxidant enzymes and reduced lipid peroxidation in the hippocampus (49). Vitamins may play a potential role in the treatment of depressive symptoms in the future, but the pathophysiological mechanism still needs to be further explored.

A review by Olivia et al. elucidated the underlying mechanism of the association between dietary fiber and depressive symptoms (25). Dietary fiber intake can alter the composition of intestinal flora and the amount of short-chain fatty acids (SCFAs) (50, 51). SCFAs inhibit histone deacetylases, which play an important role in epigenetic gene regulation (52), and high expression of histone deacetylases has been associated with depressive symptoms (53). In addition, dietary fiber may affect depressive symptoms through the activation of GPCR by SCFAs and affect depressive symptoms through intestinal flora production of tryptophan (25, 54). We also found a bidirectional association between depression and dietary patterns dominated by dietary fiber and vitamins, which may partly explain the effect of dietary fiber on depression.

There is a two-way communication between the central nervous system and the gut. Gut microbes play a key role in regulating the normal function of the gut-brain axis as well as having important effects on tryptophan metabolism and the serotonergic system (54). The intervention of some gut microbiota has the effect of serotonin synthesis and alleviates depressive symptoms (55). Second, dietary fiber may affect the level of inflammation by reducing membrane permeability through the production of short-chain fatty acids. Low dietary fiber consumption leads to increased intestinal permeability, which leads to increased endotoxin activity and induces inflammation (56).

There may be a potential association between diet and inflammation levels (57). Dietary fiber is closely associated with inflammation, and it may reduce inflammation levels by protecting the intestinal barrier and activating GPCRs (58, 59). Intestinal microbiota play an important role in the intestinal immune response. Intestinal microbial metabolite SCFAs promote innate lymphocytes and T cells to produce IL-22 through G protein receptor 41 and protect intestinal immunity (60). GPR43 and GPR41 knockout mice have defects in regulating inflammatory mediators and establishing inflammatory responses after intestinal barrier disruption. SCFAs play a role in the immune response by inducing endothelial cells to produce cytokines and chemokines, and this effect is dependent on GPR43 and GPR41 (61). Immunity has a potential regulatory effect on depressive symptoms (22). The elevated levels of inflammation have been associated with increased susceptibility to depressive symptoms (62). These results were further supported by the negative correlation between factor 3 and CRP levels. The results of our mediation analysis support that inflammation partially mediates the relationship between dietary fiber intake and depressive symptoms.

The results of previous studies are consistent with our findings that DII scores are negatively associated with depressive symptoms (63). The DII score was an indicator of overall dietary inflammation based on multiple nutrient assessments, so we used the DII score as an inflammation index. Our results also showed that the DII score was positively correlated with CRP. To ensure the independence of the DII score and individual nutrients, we made a collinearity diagnosis. The results showed that there was no collinearity between DII scores and each nutrient, which was independent. In addition, we found differences in the extent to which the DII scores and CRP levels were associated with depressive symptoms in different dietary fiber intake groups. It may be that higher dietary fiber intake can mitigate the effects of inflammation on depressive symptoms. It is necessary to further study the effects of dietary fiber intake on inflammation and depressive symptoms in future, which is of great significance to the pathogenesis and prevention of depressive symptoms.

Our study had the following limitations: (1) The cross-sectional study could not determine the causal relationship, and the dietary data were 24-h recall data, which was not representative enough. In the follow-up study, we will use image collection and a written diary to investigate dietary intake and encourage participants to record their dietary intake for a week by using mobile phone photos and written records. Considering that a very long recording time may reduce the compliance of the participants, we chose a period of 1 week as the recording time. Second, further animal experiments were conducted to explore the causal relationship between dietary fiber, inflammation, and depression. (2) In the mediation analysis model, we could not adjust for some potential confounders of the mediation results, such as sleep disorders and disease status, so the mediation model had some bias. However, we will use animal experiments in subsequent studies to prove that dietary fiber affects depression through inflammation.

## Conclusion

Our results suggest that dietary fiber intake was associated with depressive symptoms and CRP levels. The DII scores and CRP levels were not associated with depressive symptoms in people with higher dietary fiber intake. In addition, inflammation partially mediated the relationship between dietary fiber and depressive symptoms. Further prospective studies are needed to investigate the effects of dietary fiber and other nutrients on inflammation and depressive symptoms, and it is important to clarify the pathophysiological mechanisms for the prevention of depressive symptoms.

## Data availability statement

The original contributions presented in the study are included in the article/Supplementary material, further inquiries can be directed to the corresponding author.

## Ethics statement

The studies involving human participants were reviewed and approved by NCHS Ethics Review Board (ERB). The patients/participants provided their written informed consent to participate in this study.

## Author contributions

TZ was responsible for data analysis and assisting in paper writing. X-mC, Y-yZ, TX, Y-jD, and F-xG were responsible for collecting, sorting out, and analyzing data. QW, Q-fW, and M-hD were responsible for assisting in paper writing. X-tL was responsible for research design and paper writing. All authors contributed to the article and approved the submitted version.
