# Supplementary material for: Inflammation mediated the effect of dietary fiber on depressive symptoms
Source: Front Psychiatry. 2023 Jan 11;13:989492. doi: 10.3389/fpsyt.2022.989492 (PMC9874690; doi:10.3389/fpsyt.2022.989492)
Supplement: Supplementary file 1 [file Table_1.docx]

**Supplementary Table 1** Multiple linear regression between nutrient intake and PHQ-9 score in the participants.

|  | PHQ-9 | |  | PHQ-9 | |
| --- | --- | --- | --- | --- | --- |
| Variable | Standardized Coef. | *P*-value | Variable | Standardized Coef. | *P*-value |
| protein | -0.061 | **<0.019** | vitamin K | -0.011 | 0.340 |
| dietary fiber | -0.048 | **<0.010** | iron | 0.017 | 0.425 |
| PUFA | -0.003 | 0.838 | zinc | 0.032 | 0.095 |
| vitamin E | 0.039 | **0.014** | copper | -0.050 | **0.006** |
| vitamin B1 | 0.030 | 0.128 | sodium | 0.035 | 0.073 |
| total folate | -0.066 | **0.001** | potassium | -0.003 | 0.917 |
| total choline | 0.005 | 0.797 | caffeine | 0.039 | **0.002** |
| vitamin C | 0.003 | 0.841 |  |  |  |

Note: Adjustment factors include protein, dietary fiber, PUFA, vitamin E, vitamin B1, total folate, total choline, vitamin C, vitamin K, iron, zinc, copper, sodium, potassium, caffeine.
