# Supplementary material for: Inflammation mediated the effect of dietary fiber on depressive symptoms
Source: Front Psychiatry. 2023 Jan 11;13:989492. doi: 10.3389/fpsyt.2022.989492 (PMC9874690; doi:10.3389/fpsyt.2022.989492)
Supplement: Supplementary file 2 [file Table_2.docx]

**Supplementary Table 2** Relationship between depressive symptoms and dietary patterns. Standardized Coef. (*P*-value)

| variable | Factor 1 | Factor 2 | Factor 3 | Factor 4 |
| --- | --- | --- | --- | --- |
| PHQ-9 |  |  |  |  |
| Model1 | 0.002 (0.873) | -0.010 (0.258) | -0.064 (<0.001) | -0.006 (0.573) |
| Model2 | 0.021 (0.048) | 0.011 (0.287) | -0.062 (<0.001) | -0.006 (0.663) |
| Model3 | 0.009 (0.416) | 0.011 (0.297) | -0.053 (<0.001) | -0.003 (0.803) |
| Model4 | 0.023 (0.061) | 0.014 (0.245) | -0.036 (0.004) | -0.005 (0.704) |

Model 1: not adjust. Model 2 Adjusted for age, sex, race. Model 3 Adjusted for variables in model 2 + vigorous exercise, minutes of sedentary time, SBP, DBP, and BMI. Model 4 Adjusted for variables in model 3 + serious difficulty concentrating, serious difficulty hearing, serious difficulty seeing, and depression medication.
