# Supplementary material for: Inflammation mediated the effect of dietary fiber on depressive symptoms
Source: Front Psychiatry. 2023 Jan 11;13:989492. doi: 10.3389/fpsyt.2022.989492 (PMC9874690; doi:10.3389/fpsyt.2022.989492)
Supplement: Supplementary file 3 [file Table_3.docx]

**Supplementary Table 3** *OR* and 95% *CI* for depressive symptoms (moderately severe and severe depressive symptoms) according to CRP (CRP<10 mg/L) in entire group and different dietary fiber intake groups.

|  | Model 1 | Model 2 | Model 3 | Model 4 |
| --- | --- | --- | --- | --- |
| CRP (mg/L), Entire group | |  |  |  |
| Q_1_ (≤0.80) | 1.000 | 1.000 | 1.000 | 1.000 |
| Q_2_ (<0.80 - 1.70) | 0.960  (0.617 - 1.493) | 0.925  (0.593 - 1.443) | 0.875  (0.559 - 1.371) | 1.219  (0.671 - 2.212) |
| Q_3_ (<1.70 - 3.60) | 1.753  (1.198 - 2.569) | 1.660  (1.129 - 2.439) | 1.508  (1.009 - 2.255) | 1.534  (0.874 - 2.692) |
| Q_4_ (<3.60) | 2.088  (1.442 - 3.024) | 1.932  (1.328 - 2.810) | 1.674  (1.111 - 2.522) | 1.912  (1,111 - 3.293) |
| CRP (mg/L), Lower dietary fiber intake | | |  |  |
| Q_1_ (≤0.81) | 1.000 | 1.000 | 1.000 | 1.000 |
| Q_2_ (<0.81 - 1.90) | 1.274  (0.724 - 2.243) | 1.221  (0.690 - 2.159) | 1.217  (0.683 - 2.168) | 1.217  (0.663 - 2,234) |
| Q_3_ (<1.90 - 3.93) | 1.761  (1.034 - 3.000) | 1.674  (0.977 - 2.868) | 1.594  (0.907 - 2.799) | 1.495  (0.828 - 2.700) |
| Q_4_ (<3.93) | 2.256  (1.351 - 3.767) | 2,126  (1.265 - 3.573) | 2.003  (1.142 - 3.513) | 1.836  (1.015 - 3.332) |
| CRP (mg/L), More dietary fiber intake | | |  |  |
| Q_1_ (≤0.71) | 1.000 | 1.000 | 1.000 | 1.000 |
| Q_2_ (<0.71 - 1.59) | 0.998  (0.506 - 1.966) | 0.971  (0.491 - 1.920) | 0.910  (0.457 - 1.811) | 0.842  (0.407 - 1.743) |
| Q_3_ (<1.59 - 3.30) | 1.536  (0.828 - 2.850) | 1.438  (0.770 - 2.685) | 1.317  (0.690 - 2.512) | 1.261  (0.831 - 2.521) |
| Q_4_ (<3.30) | 2.447  (1.337 - 4.347) | 2.200  (1.228 - 3.942) | 2.003  (1.057 - 3.797) | 1.605  (0.814 - 3.165) |

Note: Model 1: not adjust. Model 2 Adjusted for age, sex, race. Model 3 Adjusted for variables in model 2 + vigorous exercise, minutes of sedentary time, SBP, DBP, and BMI. Model 4 Adjusted for variables in model 3 + serious difficulty concentrating, serious difficulty hearing, serious difficulty seeing, and depression medication.
