# Supplementary figures and images for: Inflammation mediated the effect of dietary fiber on depressive symptoms
Source: Front Psychiatry. 2023 Jan 11;13:989492. doi: 10.3389/fpsyt.2022.989492 (PMC9874690; doi:10.3389/fpsyt.2022.989492)

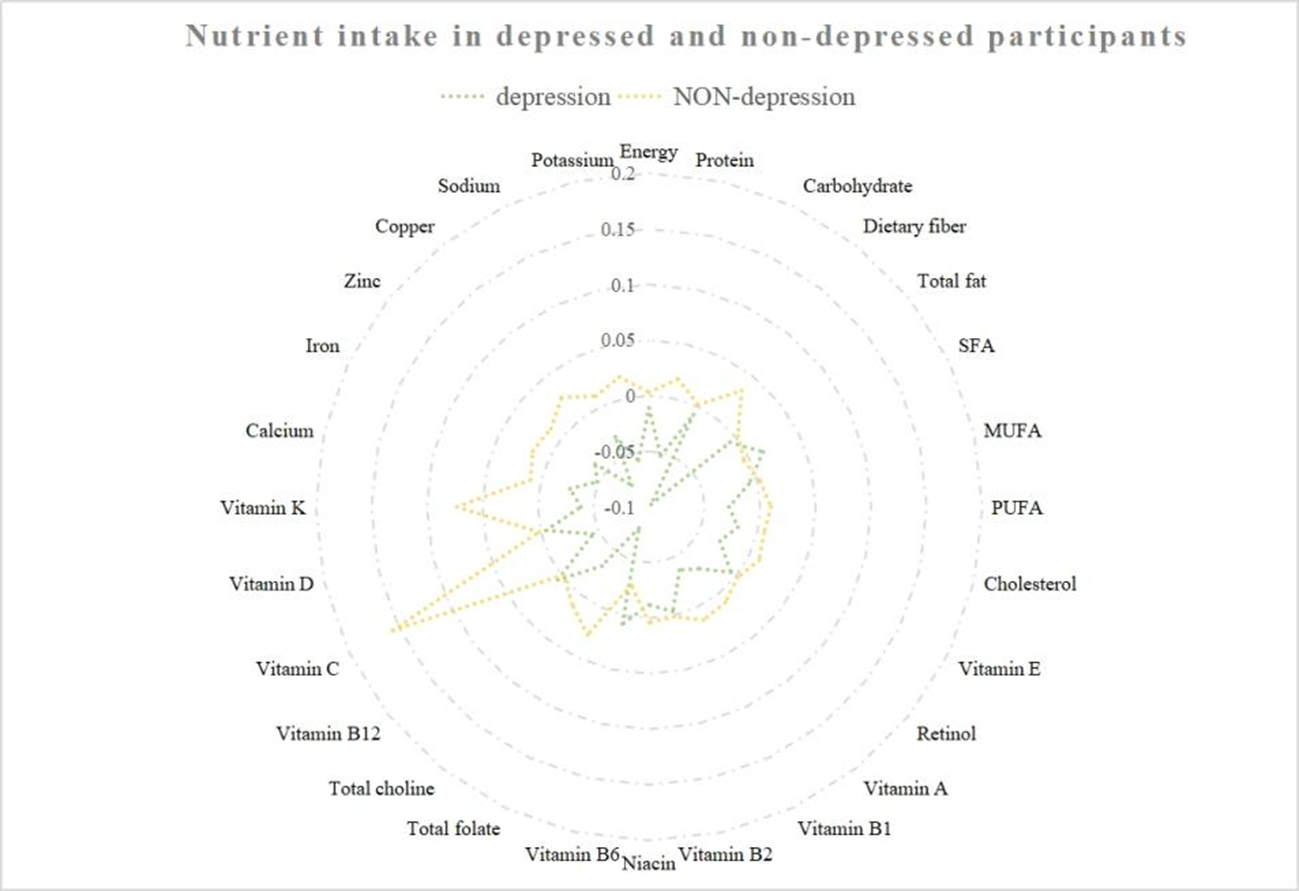

Supplement: Supplementary file 4 [file Image_1.TIF]

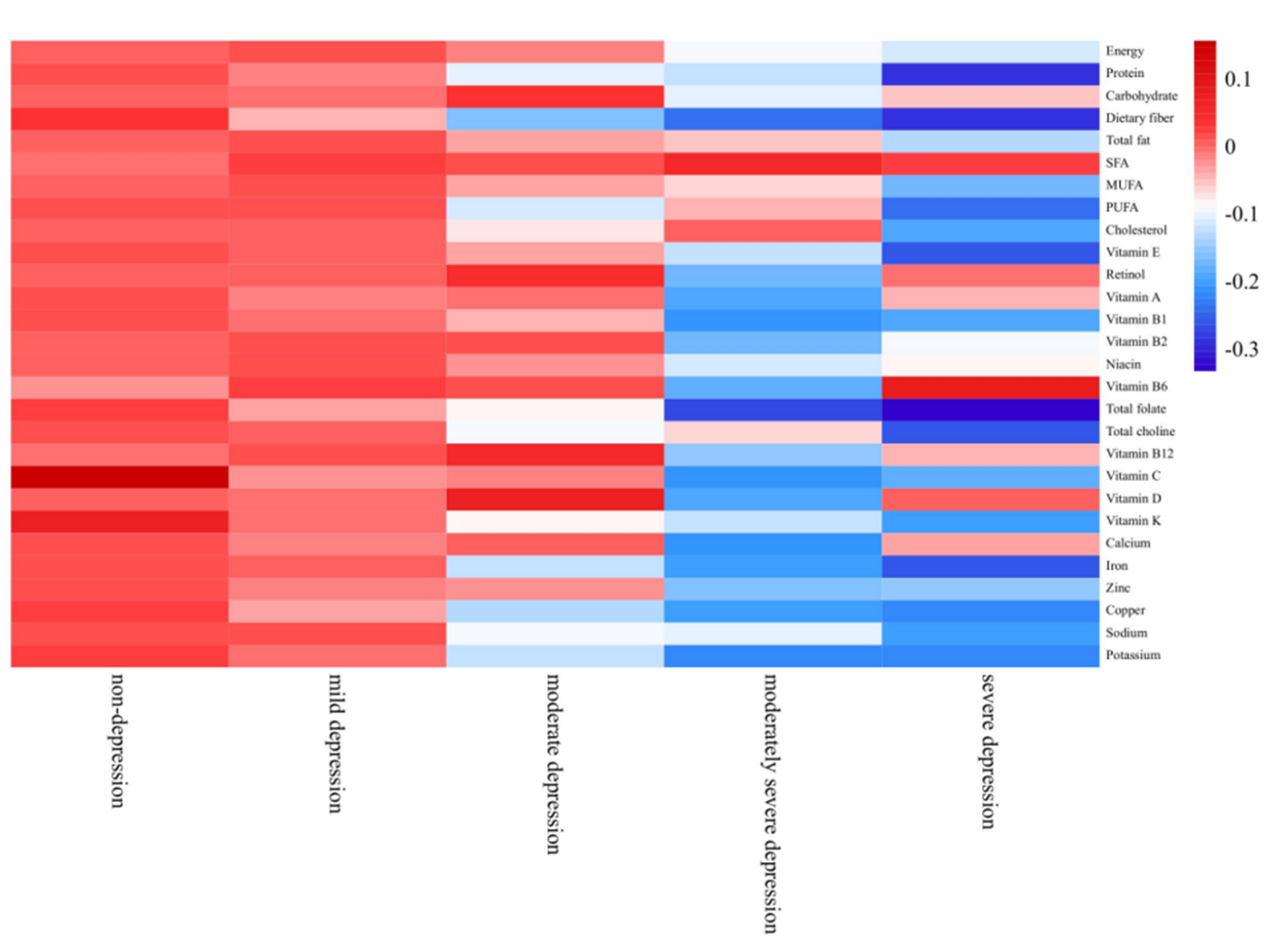

Supplement: Supplementary file 5 [file Image_2.TIF]

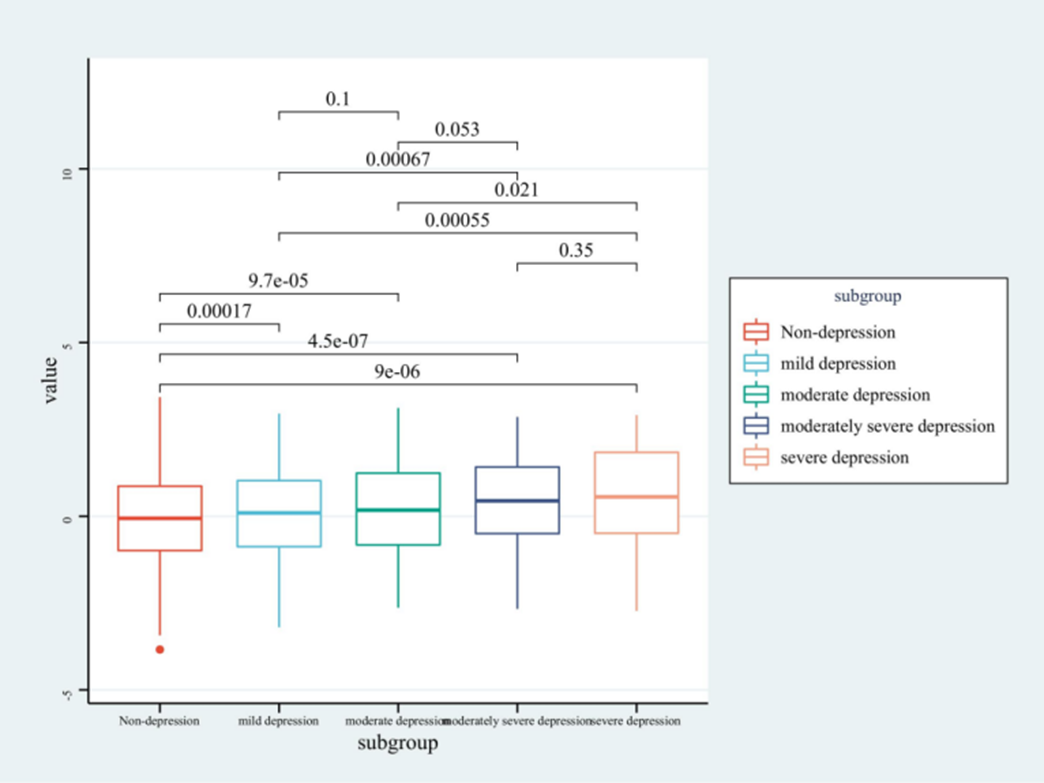

Supplement: Supplementary file 6 [file Image_3.TIF]
